# Coupling Microfluidics Data with Core Flooding Experiments to Understand Sulfonated/Polymer Water Injection

**DOI:** 10.3390/polym12061227

**Published:** 2020-05-28

**Authors:** Muhammad Tahir, Rafael E. Hincapie, Nils Langanke, Leonhard Ganzer, Philip Jaeger

**Affiliations:** Institute of Subsurface Energy Systems, Clausthal University of Technology, 38678 Clausthal-Zellerfeld, Germany; hincapie.rafael@tu-clausthal.de (R.E.H.); nils.langanke@tu-clausthal.de (N.L.); leonhard.ganzer@tu-clausthal.de (L.G.); philip.jaeger@tu-clausthal.de (P.J.)

**Keywords:** recovery factor, EOR, wettability, polymer-flooding, viscoelasticity, polymer degradation, fluid-fluid interaction

## Abstract

The injection of sulfonated-modified water could be an attractive application as it results in the formation of a mechanically rigid oil-water interface, and hence, possible higher oil recovery in combination with polymer. Therefore, detailed experimental investigation and fluid-flow analysis into porous media are required to understand the possible recovery mechanisms taking place. This paper evaluates the potential influence of low-salt/sulfate-modified water injection in oil recovery using a cross-analyzed approach of coupled microfluidics data and core flooding experiments. Fluid characterization was achieved by detailed rheological characterization focusing on steady shear and in-situ viscosity. Moreover, single and two-phase micromodels and core floods experiments helped to define the behavior of different fluids. Overall, coupling microfluidics, with core flooding experiments, confirmed that fluid-fluid interfacial interaction and wettability alteration are both the key recovery mechanisms for modified-water/low-salt. Finally, a combination of sulfate-modified/low-salinity water, with polymer flood can lead to ~6% extra oil, compared to the combination of polymer flood with synthetic seawater (SSW). The results present an excellent way to make use of micromodels and core experiments as a supporting tool for EOR processes evaluations, assessing fluid-fluid and rock-fluid interactions.

## 1. Introduction

Chemically mechanized water flooding has been studied as an enhanced oil recovery technique through sandstone core plugs and field tests [1,2,3,4,5,6,7]. Modified-water flooding is gaining much attention as an enhanced oil recovery (EOR) technique, due to its apparent lower cost and environmentally friendly characteristics, compared to other EOR methods. Modified-water is designed through the manipulation of injected brine chemistry [1,2,8,9,10]. This manipulation includes, not only the removal of some specific salts, but also the addition of active ions/salts. Such active ions are termed potential determining ions (PDI), which could disturb the established ionic equilibrium of the reservoir and contribute additional oil recovery [11]. Similarly, some ions cannot contribute to additional oil recovery termed non-PDI. Researchers report that the removal of non-PDI from injection brines could assist the production of additional oil, if a significant amount of divalent cations are present in the formation brine [2,9,12]. 

Four recovery mechanisms are reported to be the main for modified/smart water flooding or low salt flooding. Wettability alteration [13,14,15,16], multicomponent ion change (MIE) [3], clay swelling [3,13,14,16] and change in pH-value [2,17]. Special attention is given on the latest publications to either wettability alteration or interfacial viscoelasticity as the recovery mechanism. Both mechanisms are based on the ionic activities taking place among the injection brine, formation brine and rock matrix. Modified-water injection disturbs the established rock-oil-brine (R-O-B) ionic equilibrium due to the role of active ions (PDI) and helps to produce more oil. Among these active divalent ions (PDI), sulfate is the most effective ion [18]. We have previously proposed the mechanism of SO_4_^−2^ [19,20,21,22,23] for the wettability alteration in sandstone reservoirs. Similarly, from a fluid-fluid interaction point of view, sulfates can improve oil-brine interface resulting in oil phase snap-off suppression and increase the oil drop size [4,19,24]. Moreover, Mahzari and Sohrabi [25], Morin et al. [26], Sohrabi et al. [27] revealed that low salinity flooding produces more stable and viscoelastic surface at the oil-brine interface. For instance, Some researchers [26,28,29] found that this stable layer is resistant to rupture and assists the continuous oil phase transportation in the porous media, hence, contributes the higher oil recovery.

Experimental studies have reported that polymer viscoelasticity may improve oil recovery [30,31,32]. Similarly, the synergies of modified water-flood with polymer flood are expected to produce more oil as compared to single EOR technique [17]. On one hand, modified water will affect the microscopic sweep efficiency by triggering fluid-fluid and rock-fluid interactions. On the other hand, polymer flooding is expected to improve the macroscopic sweep efficiency, due to a favourable displacement mobility ratio. Hence, the hybrid process is expected to provide the combined benefits of both EOR methods [33,34,35]. Low-salinity/sulfate-modified water injection as a pre-flush is expected to change the reservoir wettability from oil-wet to water-wet and change the fluid distribution in the reservoir. In parallel with wettability alteration, fluid-fluid interactions at the oil-brine interface of detached oil are also developed. Polymer flooding after modified brine is expected to produce the redistributed oil phase easily due to improved sweep efficiency. Low-concentration polymer solutions will be required for combining with a pre-flush of low-salinity/sulfate-modified water, which will decrease the cost of EOR projects.

**Figure 1:**
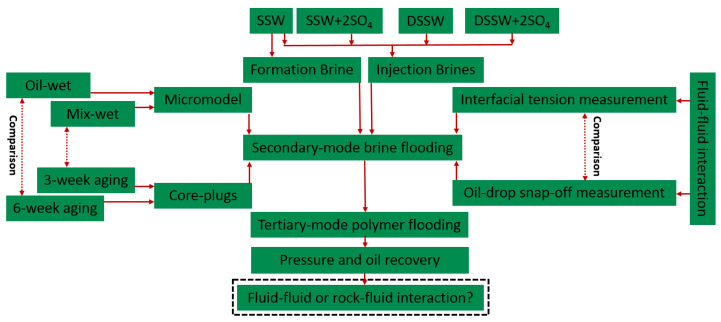
Adopted workflow considered during this study to establish the conclusive study.

This study is the extension of previous studies, which focused on the design of sulfate-modified/low-salt water-flooding for core plugs and micromodels [2,21]. This study also discusses the impact of sulfate on polymer viscoelastic behavior in porous media. This helps better understand and define the synergy role of sulfate for the pore-scale polymer viscoelastic and modified-water interfacial interaction properties on oil recovery. Hence, we evaluate the combined effect of polymer flood in combination with modified-water, for which the workflow presented in Figure 1 was adopted.

### General Approach for Evaluation

The approach helps to confirm whether the main recovery mechanism of low-salinity/sulfate-modified water injection is oil-brine interfacial elasticity or wettability alteration, or a combination of them. Furthermore, an attempt has been performed to confirm whether the combination of low-salinity/sulfate-modified with polymer flood could sweep the reservoir efficiently, resulting in higher oil recovery. The evaluation included using different fluids, porous media and experimental measurements comprising:

1. Definition, characterization and preparation of brines: One formation brine and four types of injection brine were generated. The primary approach was to prepare brines, focusing on the role of increasing the sulfate and varying the total dissolved solids (TDS) of the SSW to correlate with the impact of salinity on oil recovery.
2. Evaluation of fluid-fluid Interactions: Interfacial tension and oil-drop snap-off volume measurements were performed to investigate the ionic interaction between oil polar compounds and active ions in brine. The results of fluid-fluid interactions were incorporated to determine the possible impact on oil recovery.
3. Two-phase experiments using oil-wet and mixed/complex-wet micromodels: To understand the oil recovery contribution through interfacial viscoelastic response. Oil recovered through micromodels is mainly attributed to fluid-fluid interactions.
4. Two-phase experiments using oil-wet Bentheimer cores: To understand and define the difference in oil recovery, contributing wettability alteration and interfacial viscoelastic response.
5. Two-phase experiments, combining polymer with modified-water (micromodels and cores): To evaluate and define the synergies and benefits between modified-water and polymer flooding as the combined EOR techniques. Polymers are injected in tertiary mode through complex-wet micromodel and aged core plugs.
6. Single-phase experiments using Bentheimer cores: To evaluate the influence of sulfates (sodium sulfates) on polymer viscoelasticity and its performance in porous media based on pressure response.
7. Economic perspective analysis: Perform a basic economic exercise comparing modified-water and low salinity utilization versus the obtained recovery factor.

## 2. Materials and Methodology

### 2.1. Fluids and Chemicals

#### 2.1.1. Brines

Different salt components are mixed with deionized water to prepare the brines used in this work, as shown in Table 1. We have used deionized water in this study, which is generated using carbon filters, followed by a flow through a DI system. The resistivity at 25 °C is lower than 18 mega ohms-cm and a conductivity at 25 °C of 10 micro Siemens/cm average. The purity of all used components (i.e., NaCl, KOH, and CaCl_2_ etc.) is between 97–99% (Assay Titr.), according to salts provider, AppliChem GmbH. 

Two groups of synthetic brines (formation brine and injection brines) were prepared for this study. The brines were filtered through a 0.2-µm MF-Millipore Membrane Filter by applying 2.0 bar of N_2_ pressure to avoid any undissolved components. Injection brines were prepared to design a low-salinity/sulfate-modified water based on sulfates. The brine composition was optimized using synthetic seawater (SSW) as a base brine. SSW + 2SO_4_^−2^ represents the base brine (SSW) with a doubled amount of sulfates while SSW + 4SO_4_^−2^ indicates a quadrupled amount of sulfates. Brine optimization was further achieved by diluting (in freshwater) the SSW brine (DSSW) and SSW with the doubled sulfate amount to a tenth of its initial concentration (DSSW + 2SO_4_^−2^). The objective was to keep the TDS ≈ 5g/L to investigate the impact of low-salt brine and low-salt sulfate-modified water injection. Brine 1 and Brine 3 were used to investigate the brine hardness, salinity, and impact of sulfates on polymer viscoelasticity. Brine hardness is calculated using the proportion of divalent ions in each brine. The parameter R^+1^ (hardness) is defined according to Equation (1) by weight, as explained by Tabary et al. [36] and Tay et al. [37].

(1) $$R^{+1}=\frac{\sum \left(Divalent\;cations\right)}{\sum \left(Total\;cations\right)}$$

#### 2.1.2. Oil

Centrifuged and degassed dead crude oil (TAN is 1.15 mg KOH/g) was used for all experiments. Oil was filtered through a 5.0-µm MF-Millipore Membrane Filter to avoid solid particles and thick residue. Some oil properties are Density 0.88 g/cm^3^, 29.42° API gravity, η_o_ 23.00 mPa.s measured at 22 °C.

#### 2.1.3. Polymer Solutions

A synthetic and high molecular weight (24–28 MD) viscoelastic polymer—Flopaam 6035 S (provided by SNF Floerger from Andrezieux, France) is used to prepare diluted polymer solutions mixing 5000 ppm stock solution with five injection brines of Table 1 (Brine 1 to Brine 5), using approach adopted by Hincapie [38] and Rock et al. [17]. Five polymer concentrations (350 ppm, 750 ppm, 1000 ppm, 1500 ppm and 2000 ppm) were selected based on the desired viscosity values, subsequently injected through core plugs and micromodel. Two diluted solutions (350 ppm 750 ppm) are injected at 45 °C, through mainly core plugs. And two diluted solutions (1000 ppm and 1500 ppm) are injected in micromodel at 22 °C. Further, a 2000 ppm diluted solution was selected to investigate the polymer viscoelastic properties, while flowing through porous media. Some diluted solution resulted in viscosity half of the oil viscosity while other solutions equal the oil viscosity at 22 °C and 45 °C. Polymer concentration of 2000 ppm prepared in Brine 1 and Brine 3 is used to investigate single-phase polymer flood. The solutions were filtered to avoid fish eyes using the 5.0 µm membrane filter.

### 2.2. Fluid-fluid Interactions

#### 2.2.1. Interfacial Tension Measurements

Oil-brine interfacial tension (IFT) measurements are performed to investigate the impact of brine chemistry (monovalent and divalent ions) at the oil-brine interface. Measurements are performed using the Du Noüy ring method (Prozessor- Tensiometer KRUESS GmbH K12) at room temperature of 22 °C. The input parameters of the device are oil and brine densities and the steps for the evaluation can be described as:

- A metallic ring is placed on a fire for a few seconds to burn any organic compound if present.
- The sample holder is filled with the brine sample, and a measurement ring is inserted in the brine.
- Device calibration is performed.
- The oil phase is filled at the top of the brine phase to the marked level.
- Measurement is performed by selecting the ring movement from bottom to top (brine to oil phase).
- Towards the end of the measurement, IFT at the oil-brine interface is measured through the force experienced by a sensor attached to the metallic ring.

#### 2.2.2. Oil Drop Snap-off Volume Measurements (Fluid-fluid Interaction)

Oil-brine interfacial interactions were investigated through the analysis of oil-drop volume at the snap-off point. This approach does not provide direct measurements of interfacial viscoelasticity (G’ and G”). Rather, indirect measurement of oil-drop size at the snap-off point correlates with the interfacial interactions. Data was gathered using the following steps:

- An oil drop of 2.5 µL volume was produced through a syringe in the specific brine phase.
- A settlement time of 10 min was established for ionic equilibrium between both fluids. During this time, ionic interaction between oil polar compounds and brine divalent/monovalent ions was expected to happen at the interface.
- After 10 min, 2.5 µL of oil was further injected to increase the oil drop size.
- After a further 10 min of ionic interaction, the time between both phases was established.
- Subsequently, 2.5 µL of oil was injected to increase the oil-drop volume further.

This process continued until oil-drop snap-off happened from the needle. Oil drop experiences two opposite forces before snap-off happens. One force is buoyancy, which is an upward force due to oil density. The second force is interfacial interaction, which is a downwards force that establishes the oil-drop attachment to the needle and controls the oil-drop snap-off. Oil-drop size continues to increase in the case that the downward force at the interface is higher than the upward force. After a specific increase in drop size, buoyancy dominates the interfacial elastic force and oil-drop detachment from the needle happens.

This investigation helped to study the formation of the interfacial elastic layer at the fluid interface due to ionic reactions. The strong interfacial elastic layer is expected to produce a more significant oil-drop volume before the snap-off point. Morin et al. [26] and Mohamed and Alvarado [4] demonstrated that elastic interfacial film is found to be more stable and resistant to snap-off. This assists with stable and continuous oil flow during flooding, while limiting oil trapping in porous media, and is hence, correlated with the higher oil recovery during core flooding experiments.

### 2.3. Porous Media

Two types of porous media, micromodels and core plugs, were used for the flooding experiments to investigate fluid-fluid and rock-fluid interactions.

#### 2.3.1. Microfluidics

**Figure 2:**
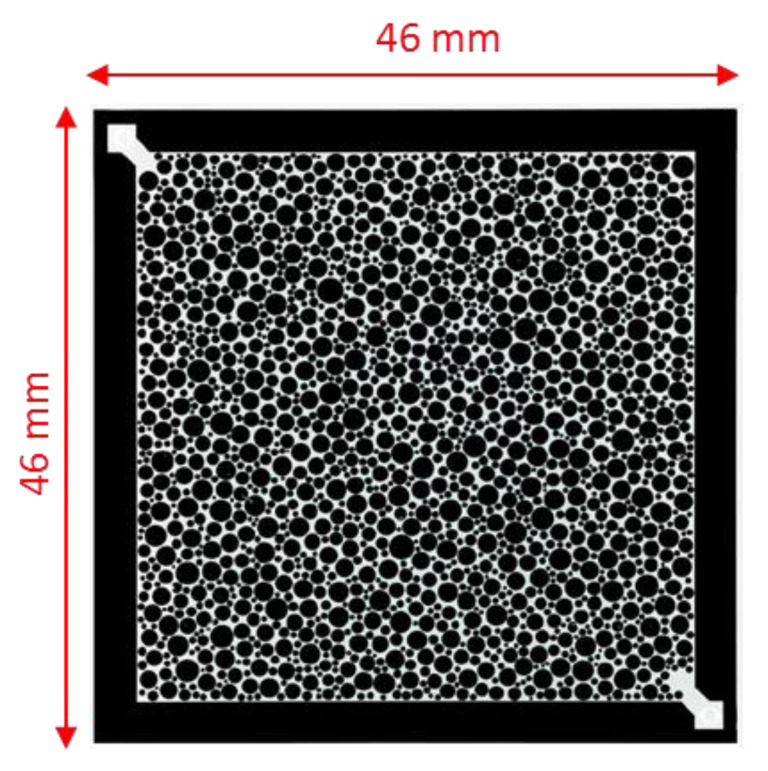
Micromodels used in this study. Scale of image is 46mm*46mm [21].

A glass-silicon-glass (GSG) micromodel was used for this study as porous media for the flooding experiments shown in Figure 2. The micromodel is an artificial structure micromodel or homogeneous micromodel, due to its random distribution of circular grains. Such micromodels have been previously used for several EOR investigations [39,40,41,42]. Figure 2 shows pore structure images and dimensional measurements of the model, and Table 2 provides porosity and permeability values.

The micromodel was chemically modified to generate two types of wettability based on the presented structures, namely oil-wet, and complex-wet/mixed-wet. The complex/mixed wettability type addresses the local variation of wettability areas, which occurs when some parts/zones are oil-wet while others are water-wet. The wettability alteration was achieved by chemisorption of fluorinated silane that was applied on the micromodels’ inner glass and silicon surfaces. Silicon and glass were initially water-wet with a contact angle (water) below 20°. After treatment, this angle was increased to 112°. The oil contact angle of the modified surface was significantly lower at 77°. The mixed-wet micromodel was obtained by fragmentary acid-induced abrasion of the coating. Wettability treatment with fluorinated silane is almost permanent. Only very strong acids and bases can attack the adsorbed layers. Silane does not decompose in the presence of crude oils as proven by a stability test at 120 °C for 8 h.

For flooding experiments, the InspIOR microfluidics-flooding rig from HOT Microfluidics was used. This is a compact experimental setup that includes injection pumps, a micromodel holder, a DSLR camera for imaging, pressure sensors (connected to the inlet and outlet of the micromodel holder), and fluid and waste reservoirs. An upgraded version of the experimental setup and components, as described by Schumi et al. [42], was used for the flooding experiments. The flooding process was performed at an injection flux of 1.0 ft/day, with corresponding injection rates included in Table 2. Bump rate injection was performed at a higher flux rate of 5.0 ft/day. Flooding experiments were performed at room temperature (i.e., 22 °C) and a system pressure of 1.0 bar (gas) with the following steps:

- Micromodel is installed into the holder and water injection is performed to remove air bubbles and pursued until the differential pressure stabilizes.
- Brine flooding is performed to measure the permeability of the model.
- Oil saturation is established through continuous and increasing oil injection rates until no further water can be produced.
- Two hours stabilization interval is provided to establish a possible ionic reaction in the model.
- Brine flooding is performed to observe the oil recovery and the pressure data.
- During the flooding process, images are gathered/captured at different time intervals and recovery analysis is performed through an imaging processing tool developed in MATLAB.

#### 2.3.2. Core Plugs

Bentheimer core plug samples were used in this study. Plugs were trimmed with an average length and diameter of 60 mm and 30 mm, respectively, and stored in the oven at 50 °C for at least three days. Porosity was measured using a Micromeritics1340 pycnometer and permeability was measured using a gas permeameter (Syroperm). Brine was injected at five injection rates (0.5. 1.0, 2.0. 5.0, and 2.0 mL/min) to measure the brine permeability.

Table 3 shows the routine core analysis parameters. Three groups of core plugs were used in this study. The first group (CG1) was selected to design low-salt sulfate-modified water (≈5 g/L) in combination with polymer flooding. Similarly, the second group of cores (CG2) was selected to investigate the impact of brine composition for slightly higher salinity, (close to SSW), in combination with polymer flooding. On the other hand, the third group (CG3) was used for single-phase polymer flooding experiments to investigate the polymer viscoelastic properties).

Two aging times were used to alter the wettability of core plugs at 45 °C, three weeks and six weeks, in order to establish attachment of polar components on the core. Skauge et al. [43] achieved the attachment of polar compounds in the Bentheimer slab through 3 weeks of aging. The aging process is expected to change cores wettability to more oil-wet. It is assumed that six weeks aging process will make core plugs oil-wet while the shorter aging process of 3 weeks will result in mix-wet to oil-wet.

A porous plate technique is used to establish the oil saturation of core plugs with a possible maximum injection pressure of 8.0 bar. Brine flooding is performed at a flux rate of 1 ft/day. To avoid capillary end effects before tertiary mode flooding, bump rate injection (2.3 ft/day) is also performed for core plugs after brine floods. Oil recovery factors and pressure profiles for bump rate injection are excluded in this study to simplify the recovery comparisons between secondary and tertiary mode flooding.

Note that, we have observed the polymer sensitivity for mechanical degradation while flowing through chokes valves before entering into the core during our previous studies [21,22]. This investigation will further provide insight into polymer viscoelastic response while flowing through porous media.

### 2.4. Rheological Measurements

Rheological experiments are performed using a Kinexus pro+ rheometer by Malvern Instruments Ltd. to characterize polymer solutions. A double gap cell stainless steel (316) geometry is used. The type of double gap geometry used is DG24/27 R0427 SS, where the bob outer diameter is 24 mm and cup inner diameter is 27 mm. Steady shear viscosity measurements are performed using the double gape geometry. Fresh polymer solutions are utilized for each measurement starting from lower to higher shear rates. Rheometer calibration was performed prior to each measurements and viscosity measurements are performed for fresh solutions (at specific temperature), in order to avoid the possible minor changes in polymer molecular structure. For further details on the detailed rheological measurement evaluation, refer to the author’s previous publications specially [44] with further details in [45,46,47,48,49].

## 3. Results and Discussions

### 3.1. Steady Shear Viscosity

The steady shear viscosity measurements can be seen as Nr. 1 through 5 in Table 4. Data are presented for the different fluids used in this work and are shown at a reference shear rate of 10 s^−1^. Important observations can be grouped as the following:

- A diluted solution of 1000 ppm resulted in viscosity half that of oil while a diluted solution of 1500 ppm has a viscosity equal to that of oil at room temperature.
- Diluted solutions of 350 ppm and 750 ppm resulted in the same viscosity due to TDS in the mixing brine. Brine 4 and Brine 5 had TDS of around 4.5 g/L while Brine 1 and Brine 2 had TDS of around 45 g/L. This predicts the significance of salt activity in designing polymer solution with the desired viscosity. One brine always has a higher sulfate content than the other.
- At a lower polymer concentration of 350 ppm, it was impossible to differentiate the viscoelastic properties of the polymer solutions based on the sulfate present [20].

We previously demonstrated [19,20] that polymers diluted in spiked sulfate brine are sensitive to mechanical degradation. Hence, we consider a seemly high polymer concentration in order to be able to further characterize the polymer viscoelastic properties (based on sulfate).

### 3.2. Fluid-Fluid Interactions

#### 3.2.1. IFT Observations

**Figure 3:**
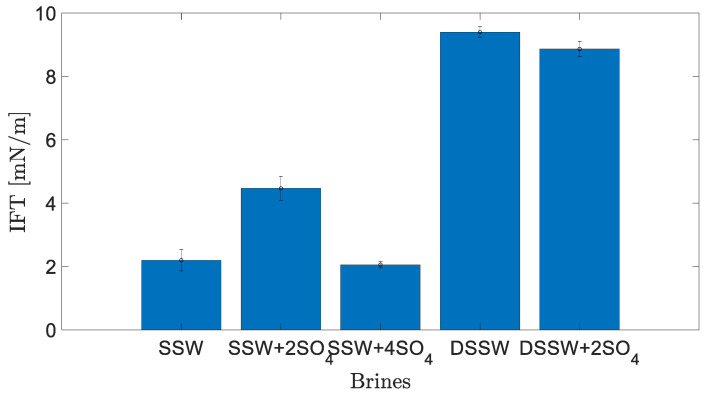
Interfacial tension (IFT) between brines and crude oil at 22 °C.

Figure 3 presents the static interfacial measurements between the brines mentioned in Table 1 and dead oil. The results show that the amount of TDS has a significant impact on IFT. The lowest values were measured for the SSW and SSW + 4SO_4_^−2^. Table 3 also shows that doubling the amount of sulfate in SSW also doubled the IFT values and that a further increase in sulfate reduced the static IFT at the fluid interface. Moreover, diluted brines resulted in the highest values of IFT for both brines. These results are in agreement with Sohrabi et al. [27], who concluded that the interfacial layer is more stable and elastic in the case of low-salt brine. This IFT response also predicts the ionic reaction between brine and oil at the interface. Active ionic interaction at the fluid-fluid interface is expected to develop a bond of divalent ions in brine and polar oil compounds (asphaltene and NAs) [25,26]. This interaction results in the development of a stable interfacial layer at the interface and hence increased IFT values.

This mechanism enhances the development of the elastic layer at the interface, which corresponds to higher recovery [4,24,26]. However, increasing the amount of sulfates in SSW by four times results in a water-in-crude oil microemulsion at the fluid-fluid interface. According to previous studies [26,50], the controlling mechanism is associated with two coalescence-suppressing interfacial barriers between fluids. Summarising the IFT response, higher values of IFT at the interface enhance the ionic interfacial properties (indirectly, elasticity), which, in turn, is expected to produce larger oil drops.

#### 3.2.2. Oil-Drop Snap-off Volume (Dynamic Fluids Interfacial) Measurements

**Figure 4:**
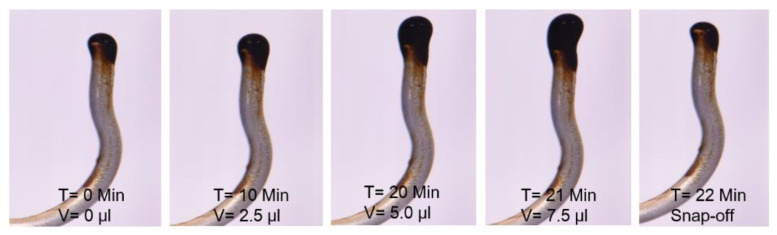
Static oil drop volume increase till snap-off point in SSW brine.

**Figure 5:**
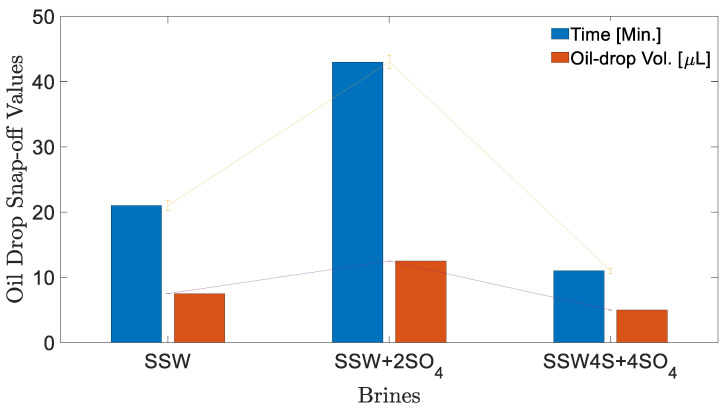
Oil drop-size analysis before snap-off for different brines at 22 °C. Time (Min.) shows time in minutes at which oil-drop snap-off happened and Oil-drop Vol. (µL) represents the oil-drop volume at snap-off point. Both connecting lines show the error bar range for measurements

Figure 4 shows the measured oil-drop volume in SSW brine. The oil drop was sustained on the needle for 21 min before snap-off, resulting in 7.5 µL oil volume. The first 20 min V-value increased by 2.5 µL per 10 min. However, between times of 21–22 min no further V value is increased, rather oil-drop snap -off happened from the needle. T = 21 Min. Figure 4 shows the oil drop image just before snap-off happened and T = 22 Min. shows the needle after snap-off point. Similarly, the oil drop volume and snap-off time was measured for two more brines, as presented in Figure 5. The interfacial response of fluids (oil-brine) appears to be in line with the results of IFT measurements. As IFT data depicted the lowest value for SSW + 4SO_4_^−2^ brine, the smallest drop size was expected for this brine.

Small drop volume was produced due to a water-in-crude oil microemulsion at the fluid-fluid interface (coalescence-suppressing interfacial barriers), which resulted in quick oil-drop snap-off from the needle. Moreover, SSW + 2SO_4_^−2^ resulted in two times the IFT compare to SSW; presumably, this is due to the generated stable layer (at the interface) due to sulfates in brine and polar oil compounds (asphaltene). The higher IFT value generated a larger oil drop of 12.5 µL. Note that it was expected that larger oil drops would be produced in diluted brine (DSSW) of Table 1. Overall, it was observed that a slightly higher IFT indicates an improved and stable interfacial layer developed at the oil-brine interface. This improved interfacial layer assists with continuous oil flow, resulting in larger oil drops (ganglia) during brine flooding and hence is expected to recover more oil.

### 3.3. Wettability Conditions of Porous Media

**Figure 6:**
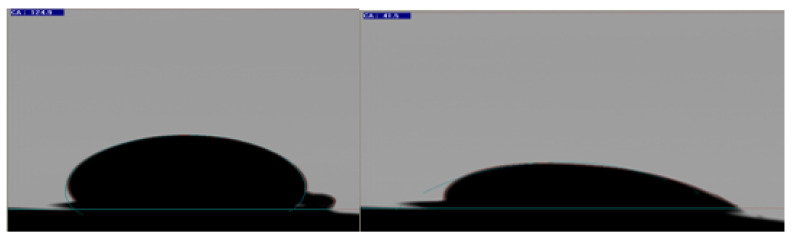
Pendant drop method contact angle measurement between oil-saturated, six weeks aged core plug and oil drop at time step 0 min (left side) and after 60 min (right side).

Contact angle data after the six-week aging process, presented in Figure 6, helped to preliminarily confirm the wettability alteration of core plugs.

Our previous study [19] provides a detailed description of contact angles for aged and unaged cores for wettability alteration. It is believed that this wettability alteration process also occurred three weeks after aging. It is assumed that for plugs with a three-week aging period, the wettability condition of mix-wet to oil-wet can be achieved. Skauge et al. [43] also proposed polar compound attachment for the Bentheimer sample.

**Figure 7:**
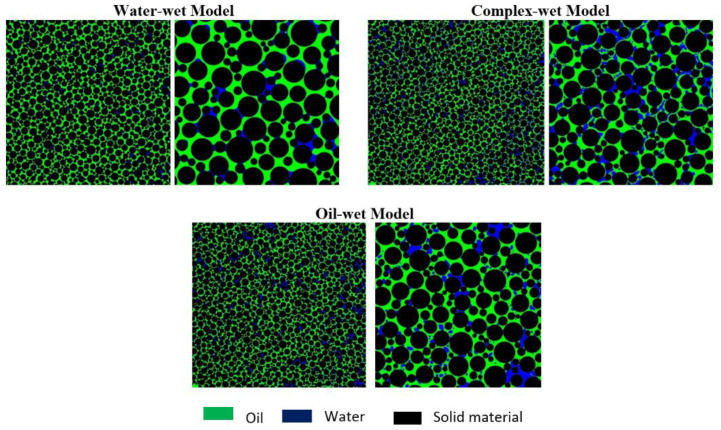
Micromodel with different wettability conditions. Left—Each wettability condition represents the micromodel (scale of 46mm*46mm); Right—Zoomed image of the bottom right corner for each micromodel (scale of 12mm*12mm).

Moreover, the wettability of the micromodel was confirmed by visual observation, through the concave/convex interface of the reservoir fluids (oil and brine) with a circular matrix structure. The concave shape of the wetting phase spreading over the rock matrix can be seen in Figure 7. However, the non-wetting phase adopted a convex shape at the fluid interface. The water-wet micromodel has the concave shape of the water phase (in blue) and the convex shape of the oil phase (in green), as presented in Figure 7. Similarly, the oil-wet micromodel has the concave shape of the oil phase (in green) and the convex shape of the water phase (in blue), as shown in Figure 7. For the complex-wet micromodel, some parts are water-wet while other parts are oil-wet. It is believed that the oil-wet model resembles the six-week aged core plugs, while the complex-wet micromodel resembles the three-weeks aged core plugs for flooding result comparisons.

### 3.4. Oil Recovery and Pressure Response for Oil-Wet Porous Media

**Figure 8:**
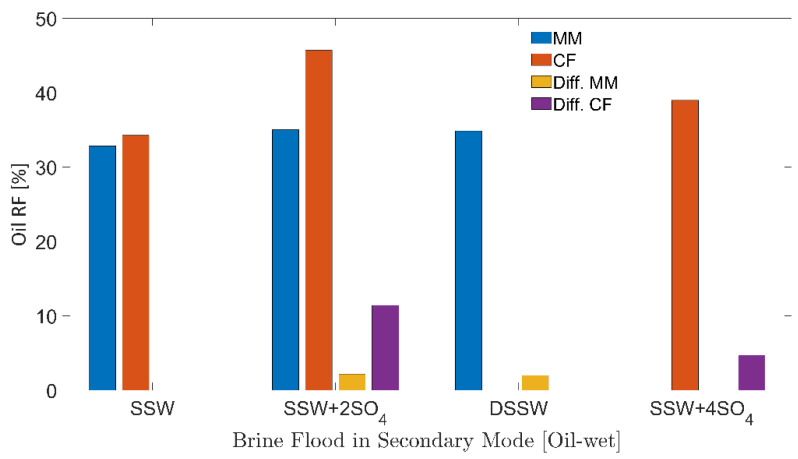
Oil recovery factors of secondary mode brines flood through oil-wet core plugs and micromodels. MM represents the oil recovery from micromodel, CF represents the oil recovery from core flood. Diff.CF/Diff.MM describes the difference in the RF of the brine flood minus the RF through SSW injection.

Figure 8 describe the secondary-mode RFs of different brine floods for six-week aged core plugs and the oil-wet micromodels. Additional recovery factor (Add. RF) in Table 5 describes the additional RF compared to the RF of SSW while Diff.CF/ Diff.MM in Figure 8 describes the difference in the RF of the brine flood minus the RF through SSW injection. Table 5 also presents the initial oil and water saturation of micromodels and 6 weeks of the aged core plugs. Both initial saturations, oil and water fall within the same range considering the specific porous media. As can be observed in Figure 8, the highest RF was achieved for both porous media when flooded with SSW + 2SO_4_^−2^.

#### 3.4.1. Oil-wet Micromodel

Brine flooding through the micromodel produced lower RFs (32–35%) for all of the brines presented in Figure 8.

There was additional oil recovery of 2%, as seen from Figure 8, for the sulfate-modified water and DSSW compared to the base brine. This 2% additional recovery can be attributed to the fluid-fluid interaction developed in the reservoir. Mahzari and Sohrabi [25], Morin et al. [26] and Sohrabi et al. [27] also reported higher oil recovery through the improved fluid-fluid interaction. Further, similar additional recovery from sulfate-modified water and DSSW supported the assumption that fluid-fluid interfacial interaction can only contribute an additional 2% oil (from the oil-wet micromodel). Significantly higher recovery from DSSW due to stronger static IFT values can be reasonably expected (IFT results), but this was not observed during the flooding process. The recovery also confirms that no additional oil was produced due to wettability alteration. It was not possible to alter the micromodel wettability to water-wet through sulfate-modified water injection or low-salt brine flooding, presumably due to the adsorption of the hydrophobic layer at the matrix. Fluid-interface confirms that the wettability of oil-wet micromodels remains unchanged after 10 PV brine flooding was performed (concave-convex contact of fluids with the matrix).

#### 3.4.2. Six-Weeks Aged Core Plugs

There was significantly higher oil recovery using SSW + 2SO_4_^−2^ for Bentheimer core plugs compared to the micromodel (Figure 8). It is assumed that this high recovery of 45.69% was obtained through the combined recovery mechanisms of wettability alteration and fluid-fluid interfacial interaction. Sulfate-modified water injection through the micromodel confirmed the 2.17% additional oil recovery through fluid-fluid interfacial interaction. Moreover, a 9.31% difference between the micromodel and core plug RFs was contributed through the wettability alteration mechanism (core plug wettability alteration to water-wet). Oil polar compounds’ attachment on the rock matrix during the aging process was not permanent, and wettability alteration was achieved through ionic interaction between the rock-oil-brine systems. The data are in good agreement with those presented for wettability alteration of core plugs through low-salt water injection or sulfate-modified water flooding [13,14,15,16]. Therefore, wettability alteration (rock-fluid interaction) in core plugs is a more straightforward approach than comparing wettability alteration in the micromodels. Hence, significantly higher oil recovery was obtained with the core plugs. The IFT and oil-drop snap-off volume measurements indicate that SSW + 4SO_4_^−2^ cannot develop a stronger fluid-fluid interaction. Further, oil recovery through SSW + 4SO_4_^−2^ flooding should be lower than that through SSW + 2SO_4_^−2^ flooding due to a weaker fluid-fluid interfacial interaction. The lower RF from SSW + 4SO_4_^−2^ is confirmed in Figure 8, which is in line with the results obtained for IFT and oil-drop snap-off volume measurements.

These results suggest that interfacial interaction as well as wettability alteration produce higher oil recovery, compared to the base SSW injection. Moreover, the primary recovery mechanism in micromodels is only fluid-fluid interfacial interaction with negligible wettability alteration. Note that, for the core plugs evaluated here, wettability alteration is the main recovery mechanism. Hence, the oil contributed from wettability alteration was much greater than the oil produced by fluids’ interfacial-interaction.

#### 3.4.3. Pressure Profiles

**Figure 9:**
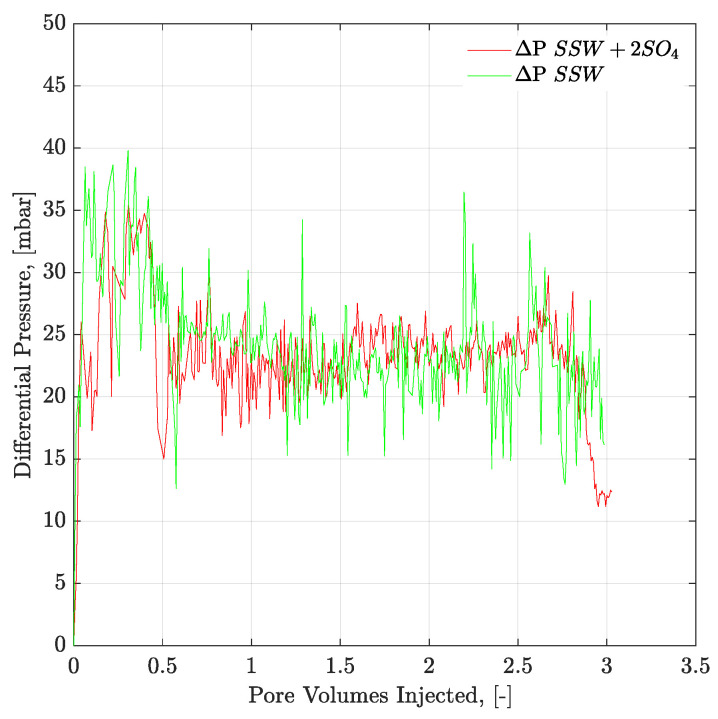
Pressure response of secondary mode brines flood through six weeks aged Bentheimer core plugs at flux rate of 1 ft/day. ΔP SSW + 2SO_4_ represents the pressure drop for synthetic seawater with doubled amount of sulfate and ΔP SSW represents the pressure drop for synthetic seawater.

**Figure 10:**
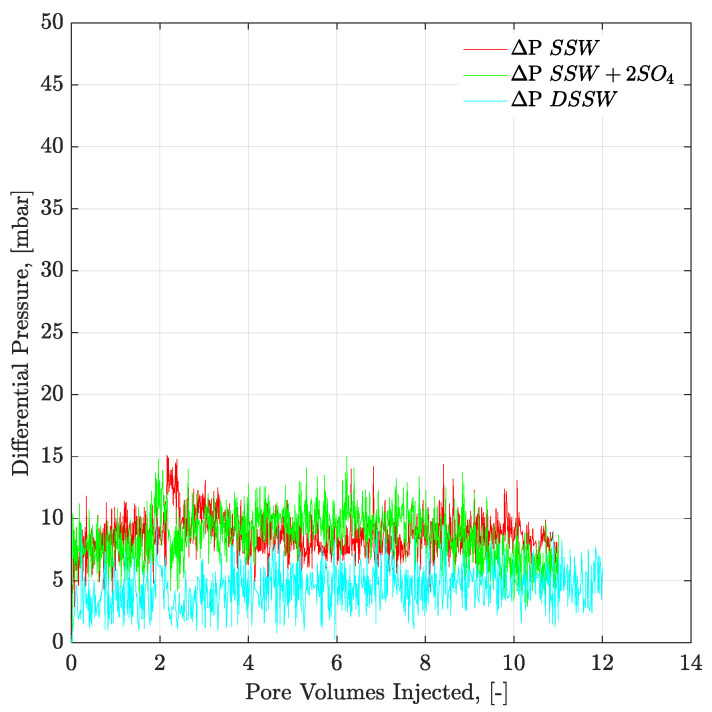
Pressure response of secondary mode brines flood through oil-wet micromodel at flux rate of 1 ft/day. ΔP SSW represents the pressure drop for synthetic seawater and ΔP SSW + 2SO_4_ represents the pressure drop for synthetic seawater with doubled amount of sulfate and ΔP DSSW represents the pressure drop for ten times diluted synthetic seawater.

Figure 9 and Figure 10 present the pressure profiles for the injected brines in the core plugs and micromodels, respectively. The pressure response through core plugs is slightly unstable with large bumps compared to the micromodel. Such bumps are expected, due to the low injection rate, compared to the oil drop movement. Further, nearly the same pressure response was observed for the injected fluids through a specific porous media (core plugs or micromodels). Brine flooding was performed at the flux rate of 1 ft/day, but core flooding resulted in almost doubled pressure values compared to micromodel flooding. One can assume that pressure difference in porous media, between Figure 9 and Figure 10, is attributed to the aging/wettability difference. However, we have confirmed the oil-wetting condition of the both porous media; the six weeks aged cores through contact angle and the oil-wet micromodel with concave/convex fluids interface. A possible reason for lower pressure drop in Figure 10 can be flux rate lower than 1 ft/day. Flux value is calculated [V=Q(A×∅)] using cross-sectional area of porous media. However, in the micromodel, fluid flow is not possible through a constant cross-sectional area it happens in the core plug. Fluid flow in the micromodel used in this work occurs in a diagonal path with injection point at one corner and production point at the opposite corner, considering a five-spot injection scheme. The diagonal flow path, according to our quantifications occurs with a varying cross-sectional area maximum in the middle and a minimum close to injection-production points. An average cross-sectional area value underestimated the flux value in micromodel and lower pressure drop can be seen in Figure 10. The half pressure obtained in Figure 10, compared to Figure 9, suggest that flux rate in the micromodel was half of the one taking place at the core plug.

### 3.5. Oil Recovery and Pressure Response for Mixed/Complex-Wet Porous Media

**Figure 11:**
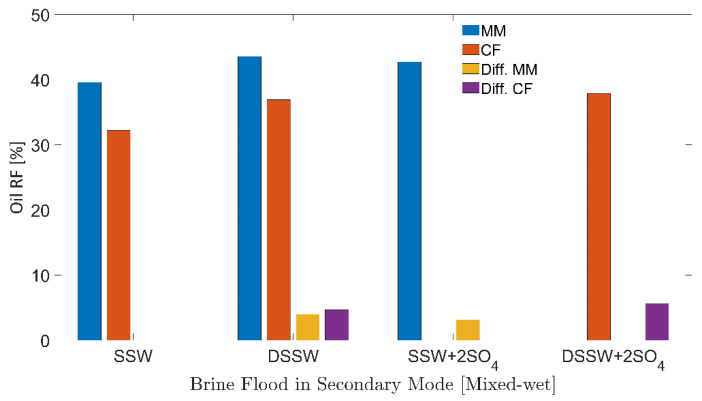
Oil recovery factors of secondary mode brines flood through oil-wet core plugs and micromodels. MM represents the oil recovery from micromodel, CF represents the oil recovery from core flood. Diff.CF/Diff.MM describes the difference in the RF of the brine flood minus the RF through SSW injection.

RFs of injected brines from three-week aged core plugs and the complex-wet micromodel are presented in Figure 11. Additional RF (Add. RF) in Table 6 describes the additional RF compared to the RF of SSW. Further initial fluid saturations, oil and connate water, are summarized in Table 6. Same values of fluids saturations can be seen from Table 6 for core plugs and micromodels, respectively. The comparison in the previous section (oil-wet system) with the mix-wet system further deepened the investigation, based on the fluid-fluid and rock-fluid interactions (wettability alteration).

#### 3.5.1. Mix-Wet Micromodel

Oil RFs for the mixed-wet micromodel are presented in Figure 11. It can be seen that both modified brines (DSSW and SSW + 2SO_4_^−2^) produced higher oil recovery (3–4%) compared to the base brine (SSW). Similar to the previous section (oil-wet), additional oil recovery was produced only through the fluid-fluid interfacial interaction at the oil-brine interface. No wettability alteration was achieved using modified water or through low-salt brine ionic activity.

#### 3.5.2. Three-Weeks Aged Core Plugs

Oil RFs of the three-week aged core plugs presented in Figure 11 were significantly lower than the RF from six-week aged plugs in Figure 8. The first reason for lower oil recovery is the difference in the wettability conditions of core plugs. During the three-week aging process, fewer polar compounds were attached to the rock matrix compared to the six-week aging period. This led to less wettability alteration during DSSW + 2SO_4_^−2^ flooding in the three-week aged core plugs. The second reason is that there was 10 times less sulfate in DSSW+2SO_4_^−2^ compared to the SSW + 2SO_4_^−2^ brine. Hence, oil recovery from the three-week aged core plugs was achieved mainly due to fluid-fluid interaction, with a weaker effect of the wettability alteration as a recovery mechanism.

#### 3.5.3. Pressure Profiles

**Figure 12:**
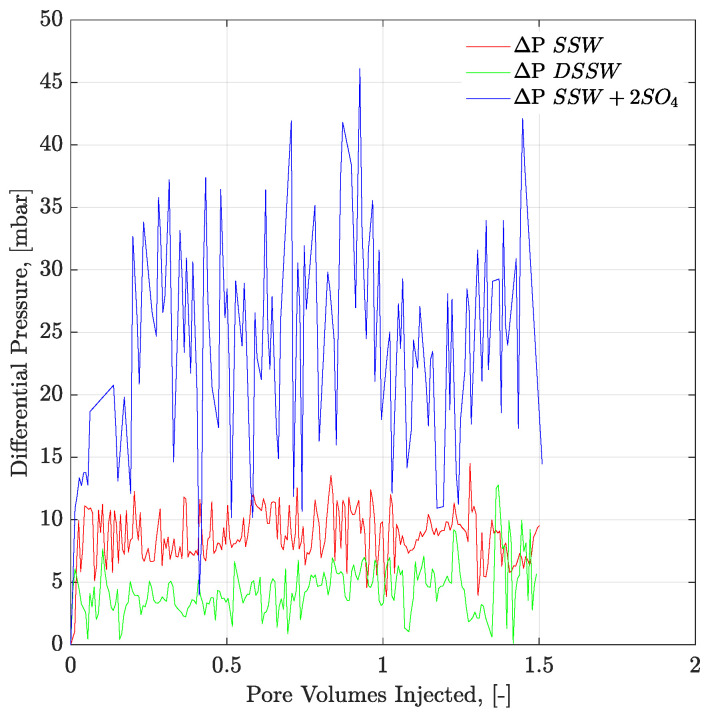
Pressure response of secondary mode brines flood through three weeks aged Bentheimer core plugs at flux rate of 1 ft/day. ΔP SSW represents the pressure drop for synthetic seawater and ΔP DSSW denotes the pressure drop for ten times diluted synthetic seawater. ΔP SSW + 2SO_4_ represents the pressure drop for synthetic seawater with doubled amount of sulfate.

**Figure 13:**
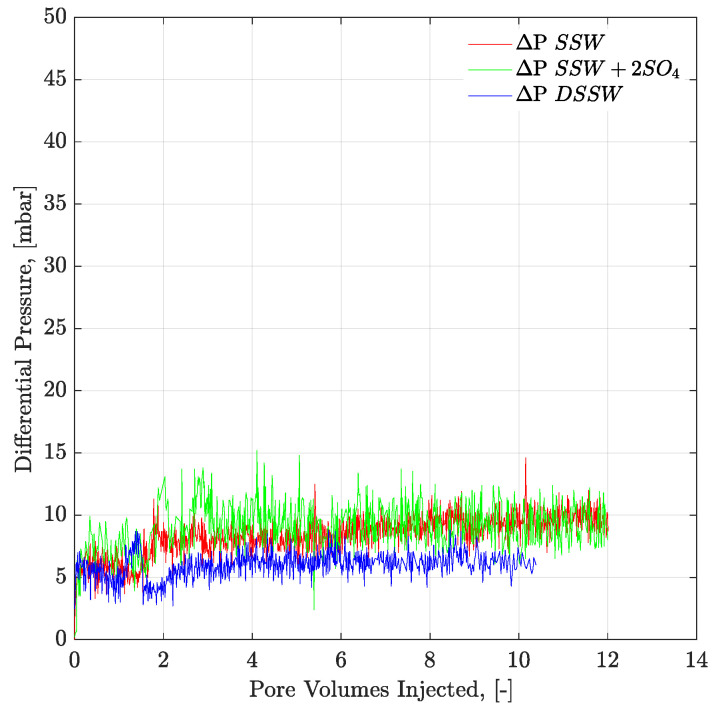
Pressure response of secondary mode brines flood through complex-wet micromodel at flux rate of 1 ft/day. ΔP SSW represents the pressure drop for synthetic seawater and ΔP SSW + 2SO_4_ denotes the pressure drop for synthetic seawater with doubled amount of sulfate and ΔP DSSW represents the pressure drop for ten times diluted synthetic seawater.

Pressure profiles of the brine floods are presented in Figure 12 and Figure 13 for core and micromodel flooding. Pressure profiles for brine flooding in micromodel are smoother than for core flooding. Core flood pressure responses with bumps, over a wide range, were also observed for oil-wet core plugs, as discussed in the previous section.

Theoretically, the difference in the additional recovery between three-week aged core flooding and mixed-wet micromodel flooding should be smaller than the difference discussed for the oil-wet system. This difference is confirmed through RFs of SSW + 2SO_4_^−2^ between CF and MM in oil-wet and complex-wet systems. The recovery difference of 2.52% in the mixed-wet system is much smaller than the difference of 9.25% for the oil-wet system. This difference in RFs emphasizes the critical role of wettability alteration as the leading oil recovery mechanism compared to fluid-fluid interfacial interaction.

### 3.6. Brine Bump-Rate Flooding

After secondary-mode brine flooding, brine bump-rate injection was performed for all of the experiments mentioned above to eliminate any capillary end effects before performing tertiary-mode polymer flooding. Through micromodel (MM), bump rate injection was performed at an injection rate five times greater than the brine rate. The core flooding was performed at a rate 2.3 times greater than the brine flooding. Oil RFs and pressure profiles for bump rate injection are excluded in this study to focus on the recovery comparison between secondary- and tertiary-mode flooding.

### 3.7. Oil Recovery and Pressure Response for Tertiary Mode Polymer Flood

#### 3.7.1. Complex-wet Micromodel

##### Polymer Viscosity Half to the Oil Viscosity (Tertiary Mode)

**Figure 14:**
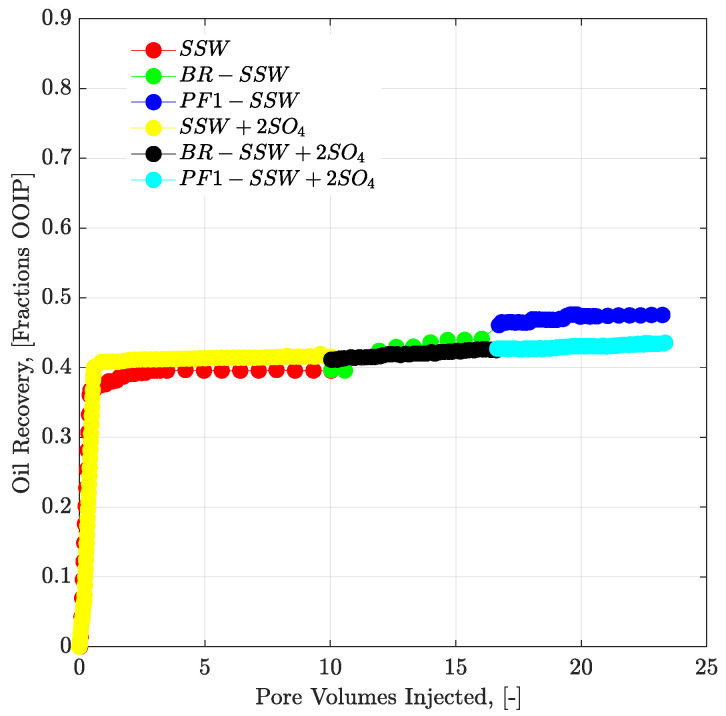
Oil recovery and pressure drop versus PV injected for complex-wet micromodel. Polymer (half to the oil viscosity) flooding in tertiary mode after the brine flood in secondary mode. SSW represents the oil recovery for synthetic seawater. BR-SSW represents the oil recovery from synthetic seawater bump rate. PF1-SSW represents the oil recovery from polymer injection prepared in synthetic seawater having viscosity half to the oil. Similarly SSW + 2SO_4_ denotes oil recoveries for synthetic seawater with doubled amount of sulfate.

**Figure 15:**
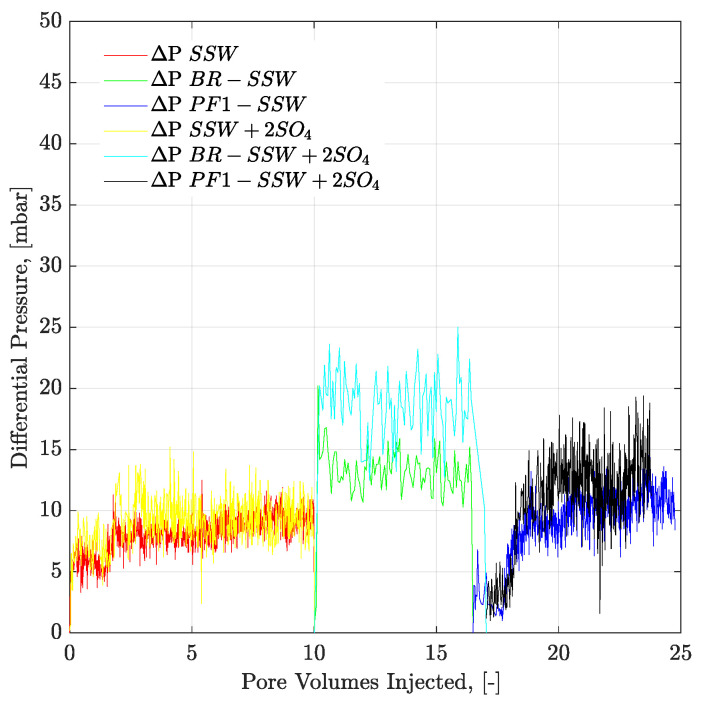
Pressure drop versus PV injected for complex-wet micromodel. Polymer (half to the oil viscosity) flooding in tertiary mode after the brine flood in secondary mode. ΔP SSW represents the pressure drop for synthetic seawater. ΔP BR-SSW represents the pressure drop for synthetic seawater bump rate. ΔP PF1-SSW represents the pressure drop from polymer injection prepared in synthetic seawater having viscosity half to the oil. Similarly ΔP SSW + 2SO_4_ denotes pressure drop for synthetic seawater with doubled amount of sulfate.

No additional oil recovery was obtained with tertiary-mode polymer flooding, as shown in Figure 14. There are two possible reasons for this:

- There is a lower polymer viscosity, compared to oil viscosity. Moreover, mechanical degradation of the polymer solution while flowing through flow lines can result in an even lower viscosity of the polymer solution than the actual polymer viscosity. Hence, polymer viscosity is expected to be less than half that of oil. Injected polymer follows the flow path of the pre-injected brine flood and cannot displace the oil due to lower aqueous viscosity.
- The pressure drop for polymer flooding is less than the pressure drop of the bump rate (pressure profiles in Figure 15. Hence, the bump rate produced additional oil due to the greater pressure drop. However, polymer flooding resulted in less of a pressure drop compared to the bump rate and hence no further oil was produced.

##### Polymer Viscosity Equal to the Oil Viscosity (Post-Tertiary Mode)

**Figure 16:**
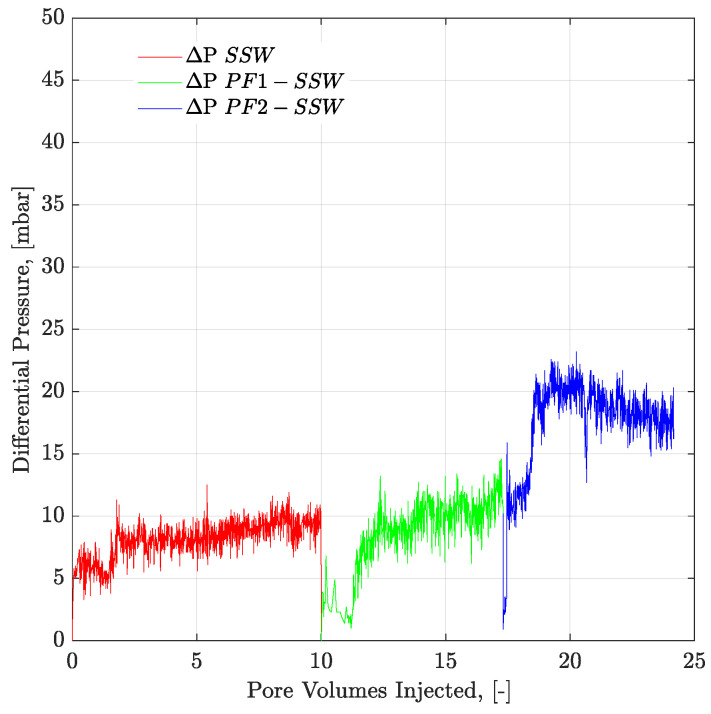
Pressure drop versus PV injected for complex-wet micromodel. PF1 (polymer half to the oil viscosity) and PF2 (polymer equal to the oil viscosity) flooding after the brine flood in secondary mode. ΔP SSW represents the pressure drop for synthetic seawater. ΔP PF1-SSW represents the pressure drop of polymer injection prepared in synthetic seawater having viscosity half to the oil and ΔP PF2-SSW shows pressure drop of polymer injection prepared in synthetic seawater having viscosity equal to the oil.

**Figure 17:**
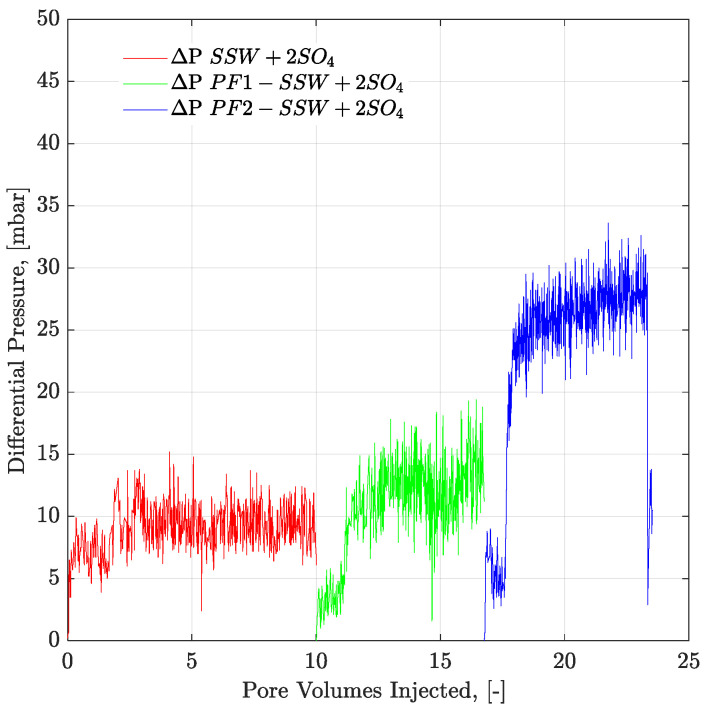
Pressure drop versus PV injected for complex-wet micromodel. PF1 (polymer half to the oil viscosity) and PF2 (polymer equal to the oil viscosity) flooding after the brine flood in secondary mode. ΔP SSW + 2SO_4_ represents the pressure drop for synthetic seawater with doubled amount of sulfate. ΔP PF1-SSW + 2SO_4_ represents the pressure drop of polymer injection having viscosity half to the oil and ΔP PF2-SSW + 2SO_4_ shows pressure drop of polymer injection having viscosity equal to the oil.

Looking at the final oil RFs of the micromodel in Table 7, the combination of brine flooding with polymer resulted in 5.71% higher recovery for SSW + 2SO_4_^−2^ compared to the combination of SSW with the polymer. As previously discussed, no wettability alteration occurred in the micromodel. This difference in oil recovery was due to the fluid’s ionic interfacial mechanism plus viscosity support of the polymer flood. This difference in oil recovery was due to the combined EOR techniques of sulfate-modified water flooding with polymer flooding. SSW + 2SO_4_^−2^ helped develop a stable ionic layer around the oil phase and produce oil ganglia inside the reservoir, while the follow-up polymer flooding helped produce these ganglia, due to improved aqueous phase viscosity. Pressure profiles of both micromodels are presented in Figure 16 and Figure 17. This post-tertiary polymer injection resulted in a higher-pressure response for polymer prepared in SSW + 2SO_4_^−2^ brine (28 mBar) compared to polymer prepared in SSW (20 mBar). This higher pressure drop, in turn, played a vital role in higher oil recovery, as seen in Table 7.

#### 3.7.2. Three-Weeks Aged Core Plugs

**Figure 18:**
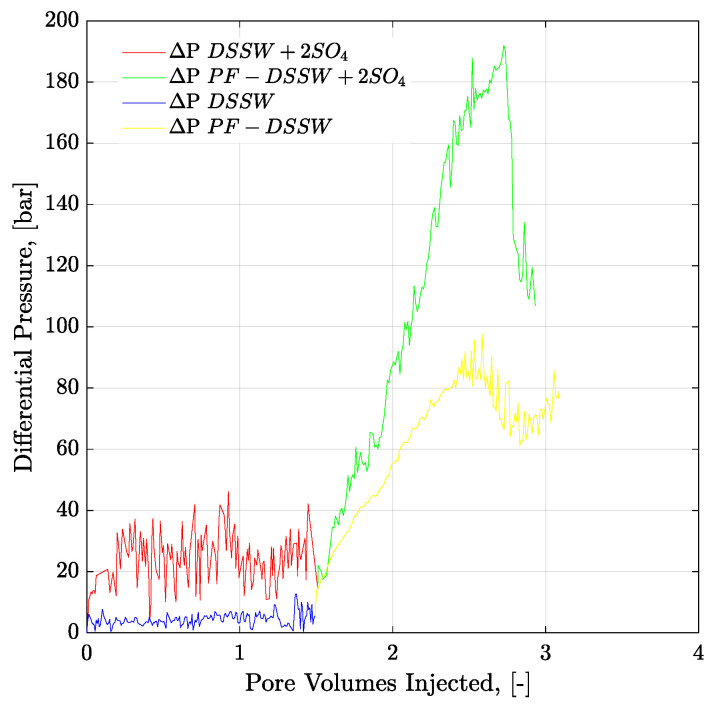
Pressure drop versus PV injected for three-weeks aged core plugs. Brine injection (≈5 g/l TDS) is performed in secondary mode while polymer flood (half to the oil viscosity) in the tertiary mode. ΔP DSSW + 2SO_4_ represents the pressure drop for ten times diluted synthetic seawater with double amount of sulfates. ΔP PF-DSSW + 2SO_4_ represents the pressure drop from polymer injection having viscosity half to the oil and prepared in ten times diluted synthetic seawater with double amount of sulfate. DSSW denotes pressure drop for ten times diluted synthetic seawater.

Figure 18 presents pressure profiles for tertiary-mode polymer flooding in three-week aged cores. Polymer solutions with half the viscosity of oil were selected for tertiary-mode injection. Looking at the RF of polymer floods for three-week aged core plugs in Table 7, 2.7% more oil was obtained from polymer flooding after DSSW + 2SO_4_^−2^. This higher recovery can be attributed to the combined effect of greater pressure drop with polymer injection combined with fluid-fluid interaction. Moreover, in comparing the final RFs after combined EOR techniques, a 3.67% higher recovery resulted from sulfate-modified water, combined with polymer flood, compared to low-salt brine combined with polymer flooding. Alteration was not the main recovery mechanism for brine flood in the three-week aging process, wettability. The main contribution of oil recovery is expected from the interfacial interaction of fluids. Pressure response for polymer prepared in the spiked amount of sulfate brines (DSSW + 2SO_4_^−2^) was higher than for the DSSW brine (almost doubled at 2.5 PV). This higher pressure response also supports higher oil recovery with polymer flooding (polymer DSSW + 2SO_4_^−2^).

#### 3.7.3. Six-Weeks Aged Core Plugs

**Figure 19:**
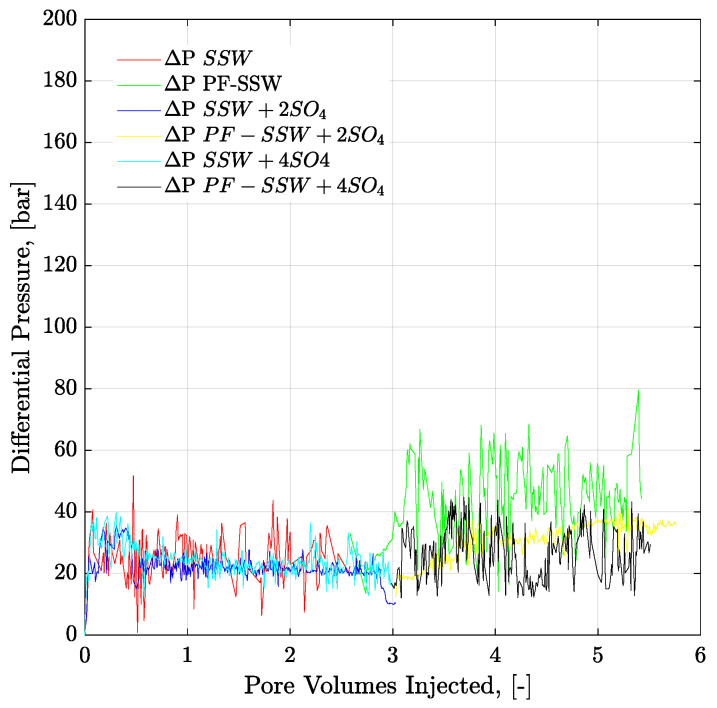
Pressure drop versus PV injected for 6-weeks aged core plugs. Brine injection (≈41–52 g/l TDS) is performed in secondary mode while polymer flood (half to the oil viscosity) in the tertiary mode. ΔP SSW represents the pressure drop for synthetic seawater. ΔP PF-SSW represents the pressure drop for polymer injection having viscosity half to the oil prepared in synthetic seawater. Similarly ΔP SSW + 2SO_4_ and ΔP SSW + 4SO_4_ denotes pressure drop for polymers in synthetic seawater with doubled amount of sulfate and synthetic seawater with quadruple amount of sulfate, respectively.

Figure 19 presents the pressure profiles of six-week aged core plugs for secondary-mode brine flooding and tertiary-mode polymer flooding. Polymers injected in the tertiary mode have viscosity half that of oil. Polymer-SSW produced the maximum amount of oil with an additional RF of 13.94%. This higher recovery was contributed due to higher ROS in the core plugs after secondary-mode SSW brine flooding. This higher amount of unflushed oil (ROS) was produced through tertiary-mode polymer flooding resulting in higher recovery. However, comparing the combined EOR effects of brine in combination with polymer flooding, sulfate-modified water (SSW + 2SO_4_^−2^) produced the highest oil recovery. The combined EOR of sulfate-modified water resulted in additional oil recovery of 6.32% compared to SSW due to strong fluid-fluid/rock-fluid interaction and follow-up higher aqueous viscosity of polymer flooding.

### 3.8. Final Recovery Factors

A summary of the final/total RFs for both porous media—core plugs and the complex-wet micromodel—can be seen in Table 7. For the data obtained, the combination of sulfate-modified water (SSW + 2SO_4_^−2^ and DSSW + 2SO_4_^−2^) always led to higher recovery compared to the base brine flood (SSW). This investigation concludes that the spiked amount of sulfate plays a significant role in disturbing the ionic equilibrium in a reservoir, which, in turn, initiates fluid-fluid and rock-fluid interactions. Comparing the final RFs obtained for SSW + 2SO_4_^−2^ flooding, combined with polymer, flood (see Table 7) rock-fluid interaction is the dominant mechanism compared to fluid-fluid interaction. Although, higher viscosity polymer was injected through the micromodels (compare to core flooding), less oil recovery was obtained. This low recovery was due to the lack of rock-fluid interaction in the micromodel. This study concludes that both mechanisms (fluid-fluid interfacial interactions and wettability alteration) are essential for higher oil recovery during low-salt or sulfate-modified water flooding, but wettability alteration is the dominating and primary recovery mechanism.

### 3.9. Single Phase Polymer Flooding

Polymer solutions are injected through stainless steel pipeline (ID = 1/8 inch) located between the injection pump and the core plug inlet. Polymer solutions are pumped at a rate of 1ft/day (the same injection rate of the core flood) to investigate polymer mechanical degradation that occur through pipes and valves. The main rationale is to define/determine in which percentage polymer degradation occurs before entering the core plugs. Degradation is therefore determined based on a comparison of the steady-shear viscosity before and after using Equation (2),

(2) $$\mathrm{Degradation}\;\mathrm{Rate}\;\left(\mathrm{DR}\right)=\frac{\eta_{o}-\eta_{e}}{\eta_{o}}\times 100$$

where, $\eta_{o}$ = viscosity of the original solution, and $\eta_{e}$ = viscosity of the degraded solution.

**Figure 20:**
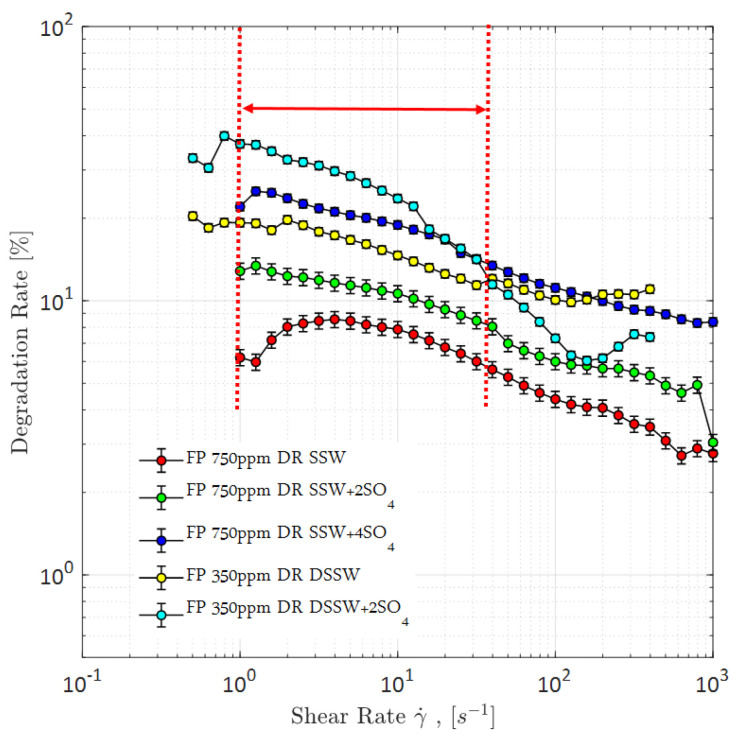
Degradation rate of Flopaam (FP) polymer solutions. Vertical red-lines represent the range of in-site shear rate in reservoir. DR SSW represents the degradation rate of polymer solutions prepared in synthetic seawater at 350 ppm and 750 ppm concentrations. Similarly DR SSW + 2SO_4_ denotes degradation rate of polymer solutions prepared in synthetic seawater with doubled amount of sulfate and DR SSW + 4SO_4_ denotes degradation rate of polymer solutions prepared in synthetic seawater with quadruple amount of sulfate.

A spiked amount of sulfate in polymer solutions makes them sensitive to mechanical degradation while flowing through flow lines, as shown in Figure 20. Polymer solutions’ viscosity is significantly decreased before entering the core plugs because an increase in the spiked amount of sulfate, in polymer solutions, increases the sensitivity to mechanical degradation, while flowing through flow lines [19,20].

However, looking at the pressure profiles through mixed-wet micromodels (Figure 15) and three-week aged core plugs (Figure 18), pressure response for polymer solution with a spiked amount of sulfate is significantly higher compared to the polymer solution in SSW. The higher pressure during polymer flooding contradicts the mechanical degradation that occurs before entering the core plugs. There can be two main reasons for the high pressure of the spiked sulfate polymer in porous media.

- The first reason is the improvement in polymer viscoelastic properties while flowing through the multiple converging-diverging geometries of the porous media. Improved viscoelastic properties cause resistance in flow due to stretching of long-chain polymer molecules and hence an increase in pressure is observed.
- The second reason is the improved oil-brine interfacial bondage developed at the brine-oil interfaces, which either develops oil ganglia or holds the water-phase attachment with the remaining oil due to a fluid’s ionic interaction. This fluid-fluid interaction indirectly narrows the flow path for polymer molecules and hence results in the higher-pressure response.

**Figure 21:**
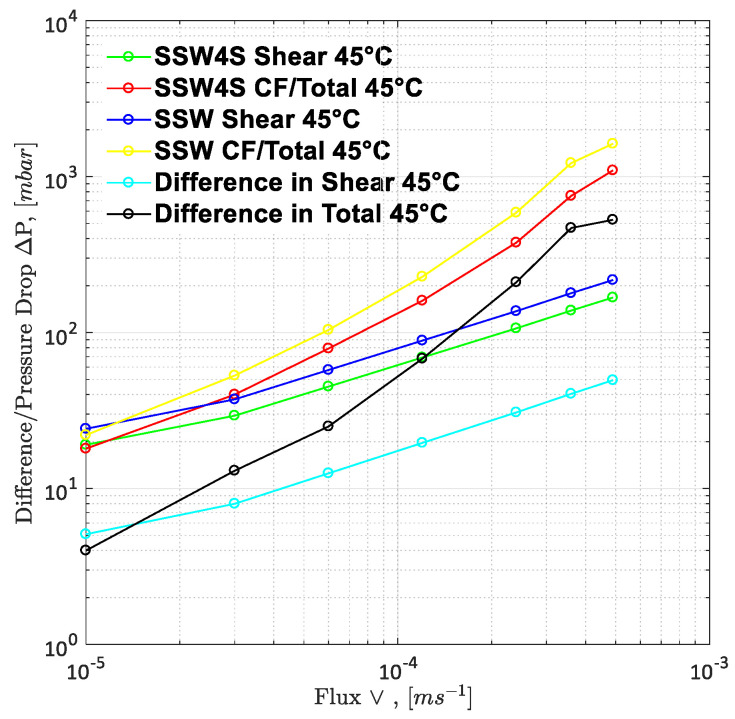
Pressure drop/Difference in pressure drop for two polymer solutions as function of injection rate. SSW4S represents the polymer solutions in synthetic seawater with quadruple sulfates and SSW denotes polymer solutions in synthetic seawater. CF represents core flood date.

**Figure 22:**
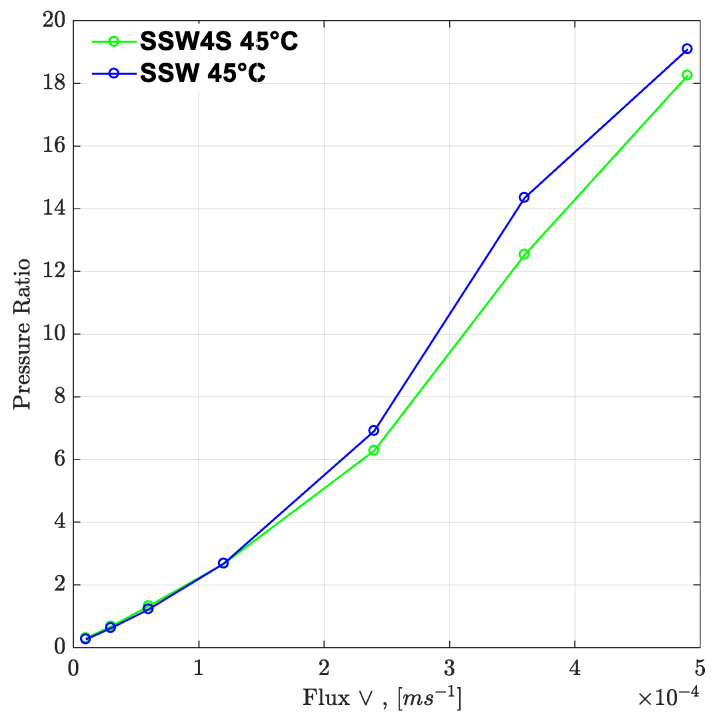
Pressure ratio as function of flux rate (two polymers). SSW4S represents the polymer solutions in synthetic seawater with quadruple sulfates and SSW denotes polymer solutions in synthetic seawater. Pressure ratio is defined as the polymer pressure drop at each flux rate divided by pressure drop for brine flood at flux rate of 10 feet/day.

To understand the leading cause of the higher pressure drop, single-phase polymer flooding was performed through the Bentheimer core plugs, with SSW and brine with four times the spiked amount of sulfate. Polymer injection (2000 ppm) was performed over a wide range of increasing flux rates of 1 ft/day to 33 ft/day, as described in Figure 21 and Figure 22. The shear pressure drop corresponds with the pressure value calculated after matching the polymer viscosity values measured by a viscometer (using Darcy’s equation) against the apparent viscosity in the core while the CF/total pressure drop corresponds with the pressure measured for core flooding. The difference in values corresponds with the values of SSW minus the values of SSW + 4SO_4_^−2^ at the same flux rate. The pressure profile for SSW polymer flooding is always slightly higher than for SSW + 2SO_4_^−2^ polymer.

Moreover, the difference in pressure drop remained constant for all flux values with the same trend line. The pressure ratio presented in Figure 21 indicates that polymer-SSW presented higher values than polymer-SSW + 4SO_4_^−2^ (flux rate higher than 1 × 10^−4^ m/s). At flux values higher than 1 × 10^−4^ m/s, the slope of both polymer solutions increased due to the dominance of viscoelastic response. However, this dominance was observed for both polymer solutions. This justifies that a spiked amount of sulfates cannot improve the viscoelastic response of polymer solutions, while flowing through porous media. Further, increased sulfates make polymer solution sensitive to mechanical degradation, which resulted in a slightly lower pressure drop compared to polymer prepared in SSW in single-phase flooding. Hence, it can be concluded that the high-pressure profiles for polymers with the spiked amount of sulfates, through micromodels and three-week aged core plugs, were due to the presence of strong interfacial layer at the oil-brine interface.

### 3.10. Economic Perspective Exercise

Low-salt/modified water is expected to be a cheaper EOR technique compared to other chemical methods. This section outlines a basic, straightforward approach for economic evaluation, focusing on the cost of modified water preparation. Modifying the injected water involves either, the removal, or addition, of specific salts, which directly affects the investment/cost incurred for a low-salt or modified water injection project. 

Table 1 summarised the various types of modified water injected in combination with polymer flooding. Here, economic evaluation is performed to select the most economical injection water recipe. Note, that this economic evaluation approximates the possible scenarios to assist with understanding the potential impacts of each technique.

#### 3.10.1. Low Salt Brines (DSSW, DSSW + 2SO_4_^−2^)

Both brines are prepared through the dilution process to reduce the TDS ≤ 5 g/L. A low-salt process occurs when the salinity of injection brine ranges 1000 to 5000 ppm. However, on a commercial scale, a significant amount of modified or diluted brine is required to execute a project. Low-salt brines can be obtained through two scenarios:

- First, they can be obtained through available resources (shallow reservoir). Low-salinity injection can be performed either by direct injection of freshwater or diluting freshwater with produced brine. Nevertheless, a significant amount of resources is essential in both scenarios. Most of the time, oil fields are located in barren places or far away from freshwater resources. Moreover, in some countries, there are restrictions on using freshwater for EOR, limiting the application of low-salt/sulfate-modified water injection at the field scale.
- Second, low-salt brines can be produced through the desalination process of the produced water. However, the desalination process is not cost-effective. A cost estimation study from Sarai Atab et al. [51] concluded that the desalination of seawater (15,000 ppm) required an investment of 11.3 GBP to obtain water with low salt (1600 ppm). Further, it required 0.8 GBP/m^3^ in operational costs. Total expenses (investment and operational cost) can significantly increase the cost of a commercial project. Qtaishat et al. [52] performed a similar economic analysis for brackish water desalination used for irrigation in the Jordan Valley. The authors concluded an average desalination investment of JD 63.5 (m^3^/h), with an average desalination cost of JD 0.38 per cubic meter.

Oil RFs of secondary-mode brine floods from Figure 11 (3-weeks aged core) indicate that without polymer flooding, it may not be economical to perform low-salt/sulfate-modified water flooding in intermediate oil-wet reservoirs (2.60% to 5.65% extra oil recovery). Nevertheless, with follow-up tertiary-mode polymer flooding, modified water with a spiked amount of sulfates can make the project economical (12.20% to 15.25% extra oil recovery).

#### 3.10.2. Spiked Sulfate Brine (SSW + 2SO_4_^−2^, SSW + 4SO_4_^−2^)

**Figure 23:**
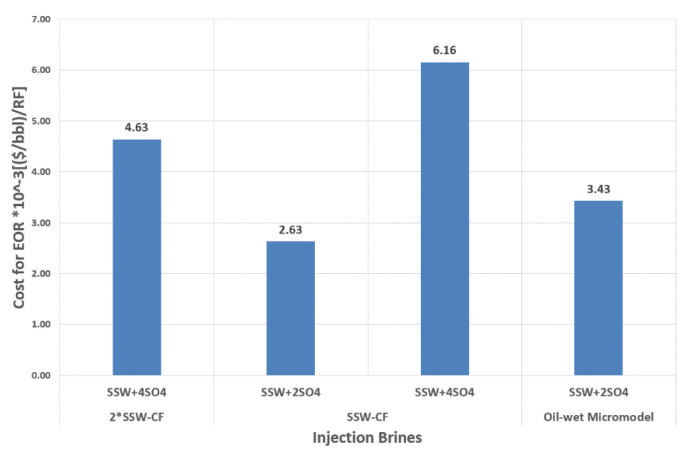
Price of the spiked amount of Sulfates in (USD/bbl.)/RF of injected modified SSW to obtain the recovery factor from Core plugs.

This group of brines was modified through the addition of sulfates in SSW. The commercial price of Na_2_SO_4_ is USD 80–120/Ton. The average price of this salt is around 0.1 USD/kg, which is much lower than the average price of polymer, alkaline, and co-solvent (3 USD/kg, 0.25 USD/kg, and 3 USD/kg, respectively [42]). The spiked amount of sulfate to design sulfate-modified water for this study is 8–16 g/L (two times and four times the sulfates in SSW). Comparing the price of Na_2_SO and the required amount of this salt, it seems attractive and commercially economical to apply sulfate-based modified water at field scale. Sulfate-modified water produced 11.42% to 17.22% extra oil recovery compared to SSW flooding, which holds significant promise. Figure 23 presents the cost of sodium sulfate to make the desired modified injection brine for the RF obtained. The expense for a doubled amount of sulfates is less than the expense for a quadrupled amount of sulfates, in terms of the oil produced. SSW-CF represents the core plug with SSW as the formation brine, while 2 × SSW-CF represents the core plug with doubled SSW as the formation brine.

## 4. Conclusions

This study facilitates our understanding of the main recovery mechanism for modified water flooding based on sulfate content, and outlines the benefit of using coupled data, obtained from core plugs and micromodel flooding. Furthermore, it confirms whether the main recovery mechanism of sulfate-modified water injection is fluid-fluid interfacial interaction or wettability alteration or a combination of these.

Based on the static interfacial tension and oil-drop snap-off volume measurements, it is clear that the doubled amount of sulfates in SSW improved the fluid-fluid interaction. This improvement in fluid-fluid interaction led to large oil drop formation in SSW + 2SO_4_^−2^ brine, which assisted with continuous oil flow, while limiting the oil is trapped in the porous media, and is hence, associated with higher oil recovery. Additionally, two-phase sulfate-modified water flooding in oil-wet and mixed-wet micromodels confirmed that the additional oil recovery can be mainly attributed to fluid-fluid interfacial interaction.

A comparison of sulfate-modified water flooding in the oil-wet core plugs with the oil-wet micromodel leads to the assumption that rock-fluid interaction is the dominating recovery mechanism in core plugs. The strong rock-fluid interaction in core plugs helped produce significantly higher oil recovery compared to the oil recovery obtained from the micromodel. Moreover, comparing the six-week aged core plug and the three-week aged core plug indicates that the oil-wetting condition of the reservoir is the primary requirement for the rock-fluid interaction.

On one hand, RFs between fluid-fluid interaction and rock-fluid interaction indicate that rock-fluid interaction is the dominating recovery mechanism in oil-wet reservoirs. On the other hand, oil recovery results show that secondary-mode sulfate-modified water injection and tertiary-mode polymer flooding resulted in the lowest ROS, hence, proposing the synergies and benefits of combined EOR techniques.

The results of single-phase polymer flooding indicate that the spiked amount of sulfates in mixing brine does not enhance polymer’s viscoelastic properties. It also proves that the pressure drop of a spiked sulfate solution is slightly less than a solution with no or fewer sulfates. Spiked sulfate polymers demonstrate a higher pressure drop in two-phase flooding due to improved interfacial (elastic) layer at the oil-brine interface developed during pre-flushed modified water. In addition, a doubled amount of spiked sulfate in SSW resulted in a cost-effective modified water process for injection in an oil-wet reservoir. No capital investment is required and no special equipment needs to be installed for mixing the small amount of sulfate in the injection brine. This recipe costs significantly less and will produce higher oil recovery. Moreover, polymer flooding in the tertiary mode after modified water flooding can recover extra oil.

## Figures and Tables

**Table 1:** Chemical composition of formation and injection brines.

Chemical Formula	Total Dissolved Solids (g/L)					
	Formation Brine	Injection/Polymer Solution Brines				
	Brine 1	Brine 1	Brine 2	Brine 3	Brine 4	Brine 5
	SSW	SSW	SSW + 2SO_4_	SSW + 4SO_4_	DSSW	DSSW + 2SO_4_
NaCl	23.97	23.97	23.97	23.97	2.397	2.39
KCl	0.80	0.80	0.80	0.80	0.080	0.08
CaCl_2_.2H_2_O	1.11	1.11	1.11	1.11	0.111	0.11
MgCl_2_.6H_2_O	11.04	11.04	11.04	11.04	1.104	1.10
SrCl₂.6H₂O	0.03	0.03	0.03	0.03	0.003	0.003
Na₂SO₄	3.93	3.93	7.86	15.73	0.393	0.78
NaHCO₃	0.27	0.27	0.27	0.27	0.027	0.02
TDS	41.15	41.15	45.09	52.95	4.11	4.50
Hardness (R^+1^)	0.13	0.13	0.11	0.09	0.13	0.11
Density (g/cm^3^) @22 °C	1.03	1.03	1.02	1.04	0.98	0.99

**Table 2:** Characteristics of micromodel and experiment used in this work.

Parameter	Glass-Silicon-Glass (GSG) Micromodel
	Artificial (Random Circles)
Porosity (%)	27.60
Brine Permeability (mD)	13,000.00
Min. Pore diameter (µm)	8.00
Max. Pore diameter (µm)	2610.00
Avg. Pore diameter (µm)	178.20
Injection Rate (µL/min)	0.30
Bump rate (µL/min)	1.50

**Table 3:** Bentheimer core plug characteristics.

	Core	L	D	phi, Φ	PV	kg	kb	Swc	Soi	Aging Time
		mm	mm	%	ml	mD	mD	%	%	
CG1	M2	59.95	29.55	23.69	9.74	2714	1964	24.60	75.40	3 Weeks
	M3	60.10	29.50	23.54	9.67	2835	1976	24.60	75.40	
	M4	60.00	29.55	24.10	9.91	2848	1608	20.60	79.40	
	M5	60.05	29.55	24.10	9.92	3029	2114	20.70	79.30	
CG2	T1	59.99	29.52	27.18	8.95	3272	2148	20.61	79.39	6 Weeks
	T2	60.11	29.36	26.53	9.18	3231	2067	15.66	84.34	
	T7	60.09	29.44	26.76	9.20	3244	1952	17.89	82.11	
	T8	59.93	29.33	26.06	8.80	3112	1970	18.67	81.33	
CG3	SP1	59.58	29.65	24.47	9.22	3131	1995	Single phase polymer flood		
	SP2	59.56	29.60	24.64	9.14	3270	2050			

**Table 4:** Polymer steady shear viscosity at a shear rate of 10 s^−1.^

Nr.	HPAM Conc.	Brine for Polymer	Polymer Viscosity	Oil Viscosity	Flooding Temperature	Porous Media	Flooding Approach
	ppm		mPas	mPas	°C		
1	350	Brine 4, Brine 5	≈3.7 (Half to oil)	8.00	45	Core	Two-phase
2	750	Brine 1, Brine 2	≈3.7 (Half to oil)	8.00	45	Core	
3	1000	Brine 1, Brine 2	≈ 9.58 (Half to oil)	21.71	22	Micromodel	
4	1500	Brine 1, Brine 2	≈ 23.58 (Equal to oil)	21.71	22	Micromodel	
5	2000	Brine 1, Brine 3	≈ 35.00 Viscoelastic study		45	Core	Single phase

**Table 5:** Oil-wet cores and micromodels floods with initial fluids saturations and oil recoveries in secondary mode brines flood.

Wettability	Porous Media	Brine Flood	Soi	Swc	RF	Add. RF
			%			
6-weeks Aged	Core plug	SSW	84.34	15.66	34.27	-
		SSW + 2SO_4_	82.11	17.89	45.69	11.42
		SSW + 4SO_4_	81.33	18.67	38.98	4.71
Oil-wet	Micromodel	SSW	85.02	14.98	32.84	-
		SSW + 2SO_4_	85.13	14.87	35.01	2.17
		DSSW	83.48	16.52	34.84	2

**Table 6:** Complex-wet cores and micromodels with initial fluids saturations and oil recoveries in secondary mode brines flood.

Wettability	Porous Media	Brine Flood	Soi	Swc	RF	Add. RF
			%			
3-weeks Aged	CF	SSW	79.40	20.60	32.22	-
		DSSW	75.40	24.60	36.90	4.68
		DSSW + 2SO_4_	75.50	24.50	37.87	5.65
Mixed-wet	MM	SSW	81.28	18.73	39.58	-
		DSSW	80.27	19.74	43.56	3.98
		SSW + 2SO_4_	80.66	19.34	42.71	3.13

**Table 7:** Oil recoveries of Core plugs and mix-wet micromodels in secondary mode brine flood and tertiary mode polymer flood.

Aging/Wettability	Porous Media	Brine Flood	Soi	Swc	Brine RF	Polymer RF	Total RF
			%				
3-weeks aging	CF	SSW	79.40	20.60	32.22	-	-
		DSSW	75.40	24.60	36.90	6.90	43.80
		DSSW + 2SO_4_	75.50	24.50	37.87	9.60	47.47
Mix-wet	MM	SSW	81.28	18.73	39.58	4.33	43.91
		SSW + 2SO_4_	80.66	19.34	42.71	6.91	49.62
6-weeks aging	CF	SSW	84.34	15.66	34.27	13.94	48.21
		SSW + 2SO_4_	82.11	17.89	45.69	8.84	54.53
		SSW + 4SO_4_	81.33	18.67	38.98	9.90	48.88

## Abbreviations and Nomenclature

BF	Brine flood	PF	Polymer flood
BR	Bump rate	PV	Pore volume
CF	Core flood	RF	Recovery factor
DSSW	Ten times diluted synthetic sea water	ROS	Remaining oil saturation
DSSW+2SO_4_	Ten times diluted synthetic sea water doubled with sulfate	S_oi_	Initial oil saturation
IFT	Interfacial tension	SSW	Synthetic sea water
IFV	Interfacial viscoelasticity	SSW+2SO_4_	Synthetic sea water doubled with sulfate
K_b_	Permeability to brine	SSW+4SO_4_	Synthetic sea water quadrupled with sulfate
k_g_	Permeability to gas	SW	Smart Water
MM	Micromodel	S_wc_	Connate water saturation
PDI	Potential determining ions	TDS	Total dissolved solids
